# Plant pathogens as introduced weed biological control agents: Could antagonistic fungi be important factors determining agent success or failure?

**DOI:** 10.3389/ffunb.2022.959753

**Published:** 2022-07-26

**Authors:** Alana Den Breeyen, Claudia Lange, Simon V. Fowler

**Affiliations:** ^1^ Manaaki Whenua – Landcare Research, Auckland, New Zealand; ^2^ Manaaki Whenua – Landcare Research, Lincoln, New Zealand

**Keywords:** mycoparasites, endophytes, classical biological control, invasive weeds, rust fungi

## Abstract

Mycoparasitic interactions are common in nature, form part of the microbiota of plants, and are considered significant contributors to fungus-fungus antagonism. Mycoparasites kill plant pathogens, protect the plant from abiotic and biotic stressors, and reduce disease incidence and severity at the plant population level. Their exploitation as biocontrol agents in agriculture is well documented. However, mycoparasites may potentially affect classical fungal biocontrol agents of invasive weed species. Classical biological control, or biocontrol, of invasive weeds involves the intentional introduction of exotic, usually co-evolved plant pathogens and insects, for permanent establishment and long-term control of the target plant. Agent establishment, effectiveness, and safety are the critical elements for a successful weed biocontrol programme. Establishment and effectiveness of agents on the invasive plant often vary throughout the invaded range with about two-thirds of weed biocontrol agents failing to suppress their target weed. There are many documented reasons why weed biocontrol agents do not establish or are ineffective when they do, and the presence and accumulation of natural enemies in the invaded range is one of them. Endophyte-enriched, invasive weeds and those forming mutualistic associations with indigenous, native endophytes could explain the lack of consistency of some classical biological control introductions. However, another variable could be factored into the mix: mycoparasitism, where one fungus parasitises another, the natural enemies of the plant’s natural enemies. In this review article, we introduce the concept of invasive weed biocontrol and the history of using plant pathogens as biocontrol agents. We discuss the success and failure of fungal agent programmes and delve into the patterns of success or failure, with a focus on the potential antagonistic role of endophytes and mycoparasites.

## Introduction

Microorganisms have many complex relationships with other organisms, with interactions ranging from mutualism and commensalism to parasitism. These behaviors foster the microbial community development through co-evolutionary processes (Weiland-Brauer, 2021). Mutualistic and commensal interactions create benefits to at least one partner and no harm to the other. However, if one partner benefits at the expense of another, the association is called antagonistic, parasitic, or pathogenic (Schulz et al., 2019). A diverse range of microorganisms likely to be associated with the invasive weed may significantly affect the pathosystem. Recent publications highlight how endophytic fungi and mycoparasites interact with and affect classical fungal biocontrol agents of invasive weeds (Kurose et al., 2012; Anderson et al., 2016; Garbelotto et al., 2019; Currie et al., 2020). In this review, the focus is on mycoparasites and endophytic fungi that reduce the effectiveness of classical biocontrol agents.

The two approaches in invasive weed biological control (biocontrol) are classical/inoculative and inundative/augmentative biocontrol. Classical biocontrol is based on the introduction of host specific exotic fungi and insects adapted to their exotic weeds for permanent establishment and long-term control (Watson, 1991; Coombs et al., 2004; Schwarzländer et al., 2018). Once introduced, if they establish and proliferate, classical biocontrol agents cause severe damage to the weed, leading to declines in biomass, reproduction, and population density (Morin, 2020). Classical biocontrol is considered the only cost-effective approach to invasive weed management across different land uses (Charudattan, 2001; Culliney, 2005; Schwarzländer et al., 2018). Inundative biocontrol is based on the mass production and release of native fungi and insects against invasive weeds. For this review we will focus solely on classical fungal biocontrol of invasive weeds.

Since the 1970s, plant pathogens have played an increasingly significant role in the field of invasive weed biocontrol (Evans and Ellison, 1990; Barreto et al., 2012). Of the 36 classical fungal biocontrol agents intentionally introduced worldwide (Morin, 2020; Winston et al., 2021), 26 are obligate biotrophs (Morin, 2020). These include the rust (Pucciniales) and smut fungi (Exobasidiales) that cannot be cultured *in vitro* and must complete their life cycle on living host plants. The success stories are well-documented and include the smut fungus *Entyloma ageratinae* R.W. Barreto & H.C. Evans released against *Ageratina riparia* (Spreng.) King & H. Rob. in New Zealand (NZ), Australia, and South Africa, bridal creeper rust fungus *Puccinia myrsiphylli* (Thüm.) G. Winter released against *Asparagus asparagoides* (L.) Druce in Australia and later found in NZ, and the gall rust fungus *Uromycladium morrisii* Doungsa-ard, McTaggart, Geering & R.G. Shivas released against *Acacia saligna* (Labill.) Wendl. in South Africa (Morin et al., 2002; Barton et al., 2007; Wood, 2012). For this reason, our review will focus primarily on intentionally introduced, obligate biotrophic fungi.

Agent establishment, effectiveness, and safety are critical elements for a successful weed biocontrol program. Although 75% of the intentionally introduced fungal biocontrol agents established post release (Morin, 2020), several rust fungi either failed to establish (e.g., *Puccinia spegazzinii* De Toni on *Mikania micrantha* Kunth), or gave slight or variable control (e.g., *Puccinia hieracii* (Probst) Jørst. var. *piloselloidarum* on *Pilosella officinarum* Vaill.) (Winston et al., 2021).

**Figure 1:**
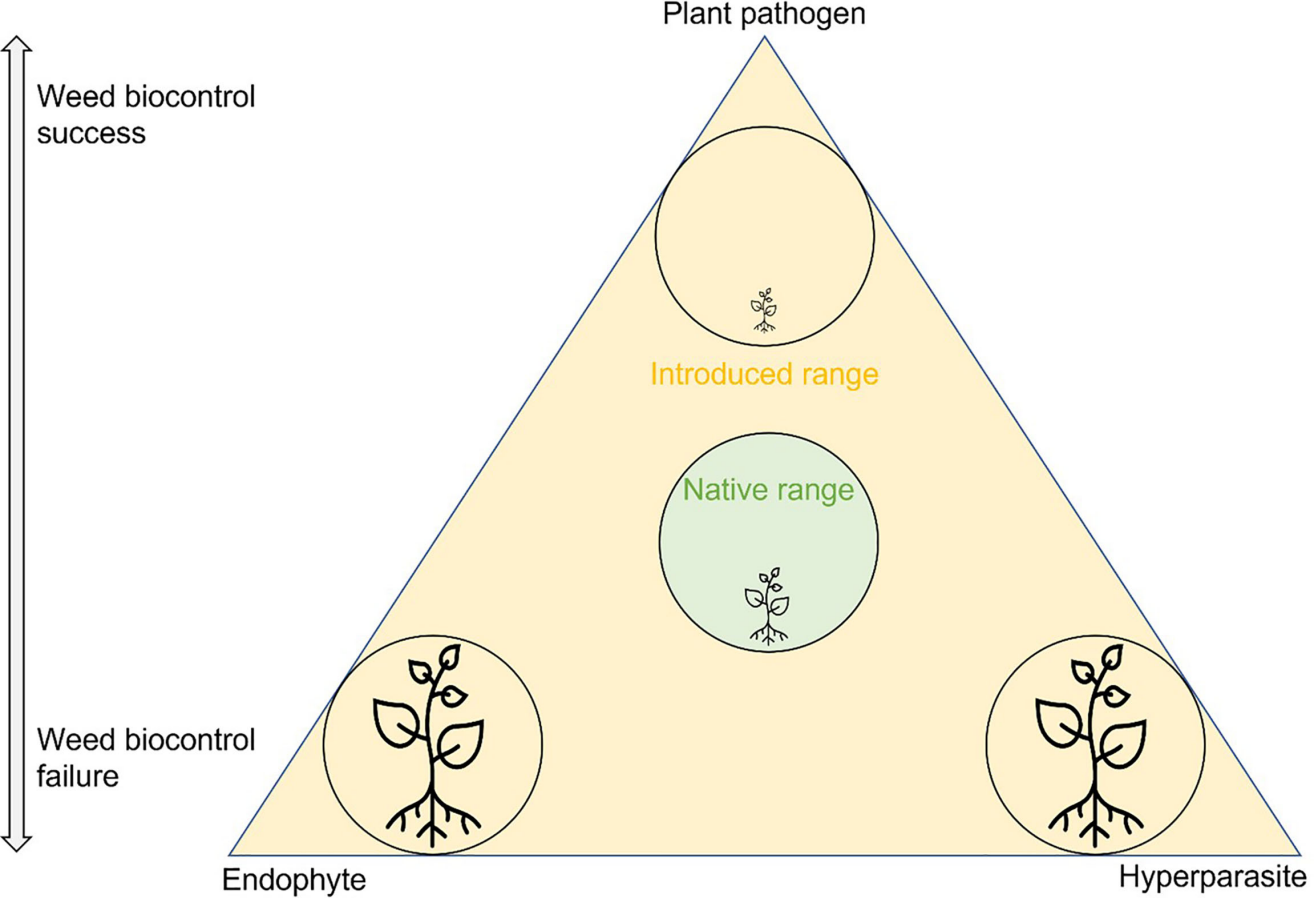
Schematic illustration of the interactions between plant pathogens and antagonists in the classical biocontrol of invasive plants. The plant, plant pathogen, and antagonist (endophyte and hyperparasite) are all potentially present in the environment. In the native range (in the green center of the triangle), the interactions are in balance, and the plant is non-invasive. In the introduced range (orange) the plant is present as an introduced exotic species. There are three scenarios: 1) The plant pathogen, introduced as a biocontrol agent, successfully suppresses the plant (top of the triangle). Its effect is stronger than that of any present endophytes or hyperparasites. 2) A protective endophyte inhibits the plant pathogen (bottom left of the triangle). Biocontrol fails, and the plant remains an invasive weed. 3) A hyperparasite inhibits the plant pathogen (bottom right of the triangle). Biocontrol fails, and the plant remains an invasive weed. Both lower parts of the triangle are examples where outbreaks of a plant pathogen have been prevented, either intentionally (biocontrol of pathogens) or unintentionally (interference with the biocontrol of weeds).

There are many documented reasons and hypotheses as to why weed biocontrol agents do not establish or are ineffective when they do, such as release or inoculum size, dispersal, population density, climate, nutritional resources, host genotypes, plant responses, competition, genetic bottlenecks, predation, and parasitism (Morin et al., 2009; Cullen et al., 2013; Seastedt, 2015; Schwarzländer et al., 2018; Winston et al., 2021). However, the effects of antagonists, such as endophytes and mycoparasites, on the establishment and effectiveness of fungal biocontrol agents are not well understood. The role of antagonists in success or failure of biocontrol needs to be evaluated to be confident about time and resources spent to design efficient biocontrol programs for invasive plant species (Schulz et al., 2019). Plants, pathogens, and antagonists interact with each other in the environment, and an imbalance of these interactions can lead either to weed invasion or to successful weed control (**Figure 1**).

There are several modes of action in which fungi can interfere with the effectiveness of deliberately introduced plant pathogens. The mechanism of action of mycoparasites and fungal endophytes are described (**Supplemental data**). Competition, antibiosis, and parasitism directly inhibit the pathogen, while plant growth promotion and the induction of the plant’s resistance to stress or pathogens limit pathogenicity indirectly. An example for a success story of antagonistic fungi are species in the genus *Trichoderma* (Mukherjee et al., 2022). Many species can better utilize nutrients than their competitors. The depletion of nutrients and the resulting faster growth enables them to exclude other microorganisms from their nutritional niche (Oszust et al., 2020). *Trichoderma harzianum* Rifai strains produce secondary metabolites that inhibit plant pathogens, such as *Rhizoctonia solan*i (Vinale et al., 2006). Cell wall degrading enzymes are enriched in many *Trichoderma* genomes. In *T. atroviride* P. Karst., genetic manipulation confirmed the role of two chitin deacetylase genes in successful mycoparasitic invasion of *Sclerotinia sclerotiorum* (Lib.) de Bary and *Botrytis cinerea* Pers. hyphae (Kappel et al., 2020). *Trichoderma gamsii* Samuels & Druzhin. and *T. afroharzianum* P. Chaverri, F.B. Rocha, Degenkolb & Druzhin. interact with maize to induce the plant’s resistance to *Fusarium verticillioides* (Sacc.) Nirenberg. The strains modulated the expression of genes that are involved in induced systemic resistance and systemic acquired resistance in plants, which led to reduced spread of the pathogen (Galletti et al., 2020).

While the intentional biocontrol of plant pathogens is the focus of this special edition, our review on unintentional interference with fungal weed biocontrol agents will be interesting for plant protection research. It contributes to a better understanding of the interactions between plant associated fungi and will improve integrated management strategies. We urge further research into the potential of fungal antagonists as biocontrol of plant pathogens, using weed biocontrol projects as model experimental systems.

## Fungal endophytes

Endophyte is the term given to all organisms inhabiting plants: arthropods, other plants, eukaryotic and prokaryotic microorganisms, regardless of whether disease or mutualism is involved (Schulz and Boyle, 2005). Fungal endophytes form part of the microbial community (or microbiome) and inhabit above- and below-ground tissues of all plants without causing visible infection or disease (Rodriguez et al., 2009). They grow inter- and intracellularly, either systematically or locally, within their hosts. Their diversity comprises species with varying lifestyles, including saprophytes, latent pathogens, mycoparasites, and entomopathogenic species (Porras-Alfaro and Bayman, 2011; Currie et al., 2014). Fungal endophytes affect plant ecology, fitness and evolution, and shape plant communities. They also change the community structure and diversity of associated organisms through increased fitness, conferring abiotic and biotic stress tolerance, increased plant biomass, decreased water consumption, or decreased fitness by altering resource allocation (Rodriguez et al., 2009).

The most common endophytic fungi are commensals, having unknown or yet unknown functions in plants. The less common ones have positive (mutualistic) or negative (antagonistic) effects on plants (Hallmann et al., 1997; Hardoim et al., 2015). Mutualistic benefits to the plant include protection against invading pathogens and herbivores (arthropods), either *via* antibiosis, induced resistance, plant growth promotion, hyperparasitism or competition (Arnold et al., 2003; Busby et al., 2016; Blackwell and Vega, 2018). A growing body of evidence supports the fact that disease-modifying fungi occur in different plant species (Arnold et al., 2003; Ganley et al., 2008; Ridout and Newcombe, 2015) and plant genotypes (Busby et al., 2013). Fungal endophytes are often described as fungal ‘bodyguards’ (Adame-Álvarez et al., 2014; Busby et al., 2016) and potentially play a key role in plant defense. It is therefore important to determine how these ‘bodyguards’ impact the efficacy of classical fungal biocontrol.

### Interference of endophytes with classical fungal biocontrol of invasive weeds

The Endophyte-Enemy Release Hypothesis (E-ERH) defines the effect of the absence or presence of co-evolved endophytic symbionts on the success of invasive plant species (Evans, 2008). This is modified from the Enemy Release Hypothesis, which defines the effect of the absence of co-evolved natural enemies on the success of invasive plants (Keane and Crawley, 2002). Endophyte-enriched invasive weeds, and those forming mutualistic associations with indigenous, native endophytes, could explain the lack of consistency of some classical biological control introductions. Non-native plants, arriving without their co-evolved natural enemies but with mutualistic co-evolved endophytes, would have a double advantage over local competitors. Non-native plants, remaining endophyte-free after arrival, would be able to allocate resources to growth and reproduction rather than to sustaining the endophyte. They would also have a distinct competitive advantage if there are no significant pressures from indigenous natural enemies or if they retain sufficient auto-defenses to overcome them. However, these endophyte-free native weeds would remain highly susceptible to co-evolved natural enemies (Evans, 2008).

Several case studies on the potential antagonistic role of endophytes on the success and failure of fungal biological control programs are discussed (**Table 1**).

**Table 1:** Fungal antagonists, mycoparasites or endophytes, associated with introduced fungal weed biocontrol agents.

Fungal biocontrol agent	Fungal antagonists	Target weed	Summary	References
*Coccodiella miconiae*	*Sagenomella dimorphica* *Cladosporium mycoparasiticum* *Redbia annulata* *Sagenomella alba*	*Miconia calvescens* (Velvet tree)	**Mycoparasites** Native range populations of this fungus are often severely hyperparasitized: a serious threat to the potential efficacy of the rust as an introduced biocontrol agent.	Seixes et al., 2005
*Colletotrichum gloeosporioides* “f. sp. *miconiae”*	*Anthostomella, Bionectria, Capnodiales, Chaetothyriales, Cladosporium, Colletogloeopsis*, *Diaporthe, Kordyana, Pestalotiopsis. Tremella*, *Verticillium*	*Miconia calvescens* (Velvet tree)	**Endophytes** Differing levels of competitive ability against the candidate agent *in vitro*.	Garbelotto et al., 2019
*Puccinia araujiae*	*Cladosporium uredinicola*	*Araujia hortorum* (Moth plant)	**Mycoparasites** Native range populations of rust in Argentina were heavily parasitized.	Anderson et al., 2016
*Puccinia komarovii* var. *glanduliferae*	*Tuberculina* sp.	*Impatiens glandulifera* (Himalayan balsam)	**Mycoparasites** Authors suggest that mycoparasitic fungus might keep the rust under control in the native range.	Tanner et al., 2015
	*Colletotrichum acutatum* *Alternaria alternata* *Cladosporium oxysporum*		**Endophytes** The three species, applied singly or in combination, lessened the impact and reduced sporulation of the rust fungus.	Currie et al., 2020
*Puccinia polygoni-amphibii* var. *tovariae*	*Colletotrichum* spp. *Alternaria* spp. *Pestalotiopsis* spp. *Phoma* spp. *Phomopsis* spp.	*Reynoutria japonica* (Japanese Knotweed)	**Endophytes** *Alternaria* and *Phoma* spp. reduced or suppressed the number of pustules produced. The *Colletotrichum* and *Pestalotiopsis* spp. had no effect while the *Phomopsis* sp. increased the number of pustules.	Kurose et al., 2012
*Puccinia spegazzinii*	Unidentified	*Mikania micrantha* (Bitter vine)	**Mycoparasites** Common in field in native range; authors suggest rust performance as a biocontrol agent might be improved when freed from mycoparasites.	Ellison et al., 2008
*Sclerotinia sclerotiorum*	60 fungal endophytes	*Cirsium arvense* (Creeping thistle)	**Endophytes** Three unnamed species, randomly selected, had either a neutral, suppressive, or positive effect on the potential biocontrol agent.	Dodd et al., 2010
*Uromyces pencanus*	*Simplicillium* sp. *Eudarluca caricis* *Cladosporium* sp.	*Nassella neesiana* (Chilean needle grass)	**Mycoparasites** *Simplicillium* sp. not obvious in the field, but severe problem in culture. *Eudarluca caricis* and *Cladosporium* sp. observed uncommonly in field.	Barton et al., 2005
*Uromyces sarothamni*	*Sphaerellopsis filum*	*Cytisus scoparius* (Scotch broom)	**Mycoparasites** Common in native range, and may reduce effect of rust on broom	Paynter, 1997 Morin et al., 2000

#### *Reynoutria japonica* (synonym: *Fallopia japonica*)

Interactions of the dominant endophytic fungi of the invasive weed *Reynoutria japonica* Houtt. (Japanese knotweed) with the rust fungus *Puccinia polygoni-amphibii* var. *tovariae* Arthur were monitored to find potential synergistic interactions in the native range in Japan (Kurose et al., 2012). The five species most frequently associated with the host plant belonged to the genera *Colletotrichum, Alternaria, Pestalotiopsis, Phoma*, and *Phomopsis*, and their effectiveness on disease development of the rust fungus was either suppressive, promotive, or neutral. *Alternaria* and *Phoma* spp. reduced or suppressed the number of rust pustules produced, while the *Colletotrichum* and *Pestalotiopsis* spp. interactions were neutral. *Phomopsis* sp. increased the number of pustules, thereby increasing its potential as a biological control agent. This is the first report of an endophyte acting in synergy with a plant pathogen, following pre-inoculation with an endophyte ahead of the rust inoculation.

#### Cirsium arvense

The possibility that endophytic fungi influenced the success or failure of *Sclerotinia sclerotiorum* (Lib.) de Bary, a potential fungal biocontrol agent for the pasture weed Californian thistle, *Cirsium arvense* (L.) Scop., in NZ was investigated (Dodd et al., 2010). Variable disease severity of the fungus led to the hypothesis that the variability was due to the presence or absence of key endophytic populations. Using both culturing and molecular techniques, the authors were able to identify which endophyte populations were present in Californian thistle plants and how much variation was present within a plant and between plants at varying distances. While the endophytes did not influence individual *C. arvense* plants, their location in the plant did, with leaves having the greatest diversity of endophytes. Selected endophytic fungi, tested to determine whether they had a significant impact on the pathogenic activity of *S. sclerotiorum* on *C. arvense*, showed that endophytes potentially influenced the success or failure of this biocontrol agent.

#### Miconia calvescens

The differential success of the fungal biocontrol agent *Colletotrichum gloeosporioides* “f. sp. *miconiae”* against *Miconia calvescens* DC., a highly invasive woody shrub in tropical oceanic ecosystems, was investigated along an elevation gradient. The role that endo- and epiphytic fungi play or contribute to the differential biocontrol success was investigated (Garbelotto et al., 2019). Quantifiable differences in foliar damage were observed across the elevation gradient, despite *C. gloeosporioides* ‘f. sp. *miconiae*’ being present at all elevations. *In vitro* testing of the endophytic fungi against *C. gloeosporioides* f. sp. *miconiae* showed differing levels of competitive ability, with higher competitive ability observed from fungi isolated at lower elevations. The foliar fungi of an invasive plant in its invaded range mediated the success of the plant’s invasion, and they concluded that this should be factored in when planning a biocontrol program (Garbelotto et al., 2019).

#### Impatiens glandulifera

In 2014, the rust fungus *Puccinia komarovii* var. *glanduliferae* R.A. Tanner, C.A. Ellison, L. Kiss & H.C. Evans was released in the United Kingdom (UK) as a biocontrol agent against *Impatiens glandulifera* Royle (Himalayan balsam), an invasive annual herb. Although the rust fungus is highly damaging in its native range, establishment success differed throughout the UK (Varia et al., 2016; Currie et al., 2020). While the patchy establishment could be attributed to variation in the plant genotype, the study aimed to determine whether other phenotypic resistance provided by indigenous foliar endophytic fungi could be involved. The endophyte communities varied between sites, showing decreased similarities between plant populations with increased distance. However, the species richness across the plant populations was uniform (Currie et al., 2020). The presence of three of the most common endophytic fungi, *Colletotrichum acutatum* J.H. Simmonds, *Alternaria alternata* (Fr.) Keissl. and *Cladosporium oxysporum* Berk. & M.A. Curtis, lessened the impact and reduced the sporulation of *P. komarovii* var. *glanduliferae.*

## Mycoparasitism

An antagonistic symbiosis between two organisms is defined as parasitism. Hyperparasites are parasites that infect other parasites and are a common phenomenon in nature. Mycoparasitism is where one fungus parasitizes another. These mycoparasitic interactions form part of the microbiota of plants and are considered a significant contributor to fungus-fungus antagonism. Mycoparasitic interactions are either necrotrophic or biotrophic (Karlsson et al., 2017). Contact necrotrophs have hypha-to-hypha interference, and invasive necrotrophs penetrate the host’s hyphae. Biotrophic mycoparasites establish a balanced relationship, with the mycoparasite growing on the living mycelium of the host fungus.

### Mycoparasitism of rust fungi

Mycoparasites of rusts are characterized by the biotrophic nature of rust fungi they parasitize and must infect in or on living host tissue (Moricca and Ragazzi, 2008). Fungi have been reported associated with rust fructifications since the early 20th century (Arthur et al., 1929). *Sphaerellopsis filum* (Biv. Ex Fr.) B. Sutton (synonym: *Darluca filum* (Biv. Ex Fr.) Castagne), *Scytalidium uredinicola* Kuhlman, J.W. Carmich. & T. Mill., *Aphanocladium album* (Preuss) W. Gams and several species of *Cladosporium*, *Tuberculina* and *Verticillium* noticeably hyperparasitise different spore stages of several rust fungi (Tsuneda et al., 1980; Wicker, 1981; Allen, 1982; Heather and Sharma, 1983; Yuan et al., 1999; Moricca et al., 2001). There are approximately 30 genera and 40 species showing mycoparasitism of rust pathogens associated with horticultural and agricultural crop species (Littlefield, 1981; Zheng et al., 2017; Sun et al., 2019), and their potential to limit the population of plant pathogenic fungi is well known. There are several studies on their use as potential biocontrol of plant diseases caused by rust fungi, including *Calonectria hemileiae* S.S. Salcedo, A.A. Colmán, H.C. Evans & R.W. Barreto against *Hemileia vastatrix* Berk. & Broome and *Alt. alternata* against *Puccinia striiformis* f. *tritici* Erikss. (Wheat stripe rust) (Barreto et al., 2015; Zheng et al., 2017).

Major outbreaks of coffee leaf rust fungus *H. vastatrix* led to a socio-economic crisis in South and Central America (Avelino et al., 2015; McCook and Vandermeer, 2015). Finding a sustainable method of disease management became a priority, providing an opportunity to utilize classical biological control against a fungal pathogen target. Both coffee (*Coffea* spp.) and *H. vastatrix* are native to Africa. The scenario was that when *H. vastatrix* spread from its native range, its natural enemies were left behind, which therefore increased its pathogenic ability. Surveys in its native range revealed unique endophytic fungi and mycoparasites that could prove useful as biocontrol agents in the neotropics. In addition, a diverse list of mycoparasites associated with the uredinia of *H. vastatrix*, previously known from central America and *via* new surveys in Brazil, represent generalist non-co-evolved mycoparasites that jumped from other fungal hosts (Barreto et al., 2015). *In vitro* and *in planta* tests of *C. hemileiae* against *H*. *vastatrix* revealed its potential as a biocontrol agent. *Calonectria hemileiae* inhibited the germination of rust urediniospores *in vitro*, while the effect of the mycoparasite *in planta* was comparable to chemical fungicide applications (Salcedo-Sarmiento et al., 2021).

Wheat stripe rust is caused by *Puccinia striiformis* f. *tritici*, one of the most important diseases of wheat worldwide, and causes large scale epidemics leading to severe yield losses under optimal environmental conditions. A novel fungal strain of *A. alternata* was identified as a hyperparasite of wheat stripe rust and was found to colonize and reduce urediniospore production and viability (Zheng et al., 2017).

These studies highlight the potential usefulness of mycoparasites in controlling plant diseases. However, there are very few records in the literature on the potential negative impact of mycoparasites of fungi released as biocontrol agents of invasive weeds, and these are mostly anecdotal.

### Anecdotal evidence of mycoparasite interference in classical fungal weed biocontrol

Earlier we discussed the E-ERH hypothesis, where plants arriving in exotic situations without their co-evolved natural enemies (endophytes) have increased levels of fitness compared with native species. Because of the absence of their endophytic bodyguards in the native range, they stay vulnerable to their co-evolved natural enemies (Evans, 2008). However, another variable could also be factored into the E-ERH: the natural enemies of the plant’s natural enemies. Mycoparasitic fungi are one natural enemy of rusts that have been observed attacking rust fungi used as biocontrol agents for invasive weeds (**Table 1**). These observations have been made either in the native range of the weed, or in the introduced range of the weed after the rust has been introduced as a biocontrol agent.

#### Cytisus scoparius

*Cytisus scoparius* (L.) Link is a widely commercialized ornamental perennial shrub found in temperate and subtropical regions of the world. It is considered a serious invasive weed in temperate areas of the United States, Canada, Hawaii, Chile, Argentina, the eastern halves of both islands of NZ, Australia (including Tasmania), India, Iran, Japan and South Africa (CABI, 2019). As an aggressive fast-growing invader, it has the capability to form dense impenetrable monospecific stands that create fire hazards, degrade native grasslands, forests, and agricultural lands, and prevent regeneration of natural forests and prairies.

In 1995, the rust fungus *Uromyces pisi-sativi* (Pers.) Liro was discovered infecting broom stems in Europe, causing severe branch dieback (Paynter, 1997; Morin et al., 2000a). Initially considered as a potential biocontrol agent against broom in Australia and Europe, the fungus was found to be non-host specific as it developed extensively on tagasaste (a fodder plant used particularly in Western Australia). In the native range of *C. scoparius*, the mycoparasite *S. filum* is associated with the rust fungus, and uredinia infested with the mycoparasitic fungus had to be discarded before inoculation. The infestation led to reduced amounts of rust inoculum that was available for the biocontrol program (Morin et al., 2000a).

*Uromyces pisi-sativi* was later found to be widespread throughout the range of broom in Australia (Morin et al., 2006). A 2013 survey of the broom biocontrol agents at 57 sites in the Alpine National Park (Victoria, Australia) showed that over half the sites (58%) were infected with broom rust. Infection levels varied, from light infection levels having negligible impact on host fitness to locations with high to severe infection levels having a debilitating effect of the host (Pascoe et al., 2014). However, there is no information on whether mycoparasites were found associated with the agent, and to what extent they contributed to the variation in field infection.

#### Miconia calvescens

*Miconia calvescens*, an invasive tree in French Polynesia, New Caledonia, Hawaii, and Australia, is listed as one of the 100 world’s worst invasive alien weed species (GISD, 2022). The fungus *Coccodiella miconiae* (Duby) Hino & Katumoto was identified as having great potential as a biocontrol agent against *M. calvescens*. While the fungus is found on *M. calvescens* throughout the year, disease severity varies from being almost undetectable to heavy infections causing deformation and chlorosis of infected shoots (Seixas et al., 2007), and the difference in virulence in the field could not be explained.

In its native range, the stroma of the fungus is often severely hyperparasitized by mycoparasites commonly observed on *C. miconiae*, including *Sagenomella, Cladosporium, Redbia, Corynespora, Paranectriella, Paecilomyces, Periconiella*, and *Pleospora* spp. (Seixas et al., 2005). Two *Sagenomella* species were found to be the most common and most damaging hyperparasites of *C. miconiae*, which was a serious barrier to keeping the potential biocontrol agent alive on plants. To what extent these mycoparasites would limit the agent efficacy and potential damage of *C. miconiae*, if released as a biocontrol agent, is not clear (Seixas et al., 2007).

#### Impatiens glandulifera

In addition to the effect of foliar endophytes on the severity of the rust fungus *P. komarovii* var. *glanduliferae*, an unusual mycoparasite was associated with the aecial stage of the rust fungus on *I. glandulifera* in the Himalayas. The *Tuberculina* sp. is systemic in the plant and completely replaces the aecial cups (Tanner et al., 2015). The authors suggest that the mycoparasitic fungus keeps the *P. komarovii* var. *glanduliferae* under control in its native range, and in the absence of these co-evolved mycoparasites in the invaded range, the rust fungus experiences increased fitness (Tanner et al., 2015). Therefore, the selection of mycoparasite-free classical fungal biocontrol agents is important for increasing the efficacy of the agents.

#### Araujia hortorum

*Araujia hortorum* Brot. (Moth plant), a climbing vine, is invasive in NZ and considered a minor weed in Australia. As moth plant has the potential to cause significant environmental damage in NZ, permission to import and release the rust fungus *Puccinia araujiae* Lév. as a biological control agent of moth plant was approved (Anderson et al., 2010).

Implementation of the biocontrol agent could, however, be compromised by a mycoparasite, *Cladosporium uredinicola* Speg., found associated with the rust fungus in Argentina (Anderson et al., 2016). Attempts to establish a mycoparasite-free culture in the laboratory through superficial disinfection and multiple sequential inoculations were only partially successful: despite high levels of mycoparasitism, the rust fungus still caused damage and defoliation of host plants in the field (Anderson et al., 2016). Interestingly, *C. uredinicola* has been recorded from *Puccinia coprosmae* Cooke on *Coprosma macrocarpa* Cheeseman in NZ (Bensch et al., 2012) and *Melampsora laricis-populina* Kleb. on *Populus* sp. (Heuchert et al., 2005). If the biocontrol agent is introduced into NZ, these mycoparasites could potentially infect *P. araujiae* teliospores, thereby limiting the impact of the agent in the field.

#### Nassella neesiana

Native to South America, *Nassella neesiana* (Trin. & Rupr.) Bark. (Chilean needle grass) is a serious grassland weed in NZ, a ‘Weed of National Significance’ in Australia, and among the most troublesome weeds threatening native vegetation worldwide (Giordano and Anderson, 2021).

After detailed field surveys, the rust fungus *Uromyces pencanus* (Dietel & Neger) Arthur & Holw. was identified as a potential biocontrol agent against *N. neesiana* in NZ. A mycoparasite identified as *Simplicillium* sp. was found associated with the rust in the glasshouse in Argentina (Anderson et al., 2010). The mycoparasite was successfully eliminated by storing the spores in the freezer. However, several species of mycoparasitic fungi are associated with rust fungi in NZ, including *S. filum*, *Tuberculina*, *Lecanicillium*, and *Cladosporium* species, and it is unknown whether these mycoparasites could potentially impact the agent in the field.

#### Mikania micrantha

*Mikania micrantha* is a perennial, creeping vine, with the common name ‘mile-a-minute weed’ in its invaded range of India and the Pacific region (GISD, 2022a). The neotropical rust fungus *Puccinia spegazzinii* was released as a classical biocontrol agent against the weed in India in 2005 and Taiwan and Papua New Guinea in 2008. The rust fungus was found to be more pathogenic on glasshouse-grown populations of the weed from its invasive range, than on the original host biotype from which it was isolated (Ellison et al., 2004). Several pathogen pathotypes were eventually considered for introduction into Asia and the Pacific as each rust pathotype infected only a selected number of host genotypes.

The limiting factors were ascribed to the absence of pathogen selection pressure on the plants in the exotic range that led to a loss of resistance, or to the loss of co-evolved endophytes and their protection from natural enemies in the invasive range (Ellison et al., 2008). Mycoparasites were commonly found in the field in the native range. The authors postulated that the rust fungus, without its co-evolved natural enemies (i.e., mycoparasites), and under different environments, should have had a greater impact on *M. micrantha* in the invaded range compared with the native range.

#### Acacia saligna

In the mid-1800s, *A. saligna* was introduced into South Africa from southwestern Australia for dune stabilization in several coastal areas. It was regarded as the most troublesome invasive alien weed in the Cape fynbos floristic region of South Africa (Morris, 1991). In 1987, the gall-forming rust fungus *Uromycladium morrisii* Doungsa-ard, McTaggart, Geering & R.G. Shivas was released in South Africa as a biocontrol agent for *A. saligna.* The pathogen was actively released from 1988 to 1996 throughout the Western Cape province and the southwestern region of the Eastern Cape province, resulting in the establishment of the rust fungus throughout the range of the weed in South Africa (Morris, 1991).

Post-release evaluations revealed that several fungi, including *Trichothecium roseum* (Pers.) Link : Gray, *Tuberculina* sp. and *Verticillium* sp. caused considerable damage to the galls (Morris, 1997). Teliospore production was significantly reduced by the *Tuberculina* sp., which often covered up to 60% of the surface area of many rust galls. The *Verticillium* sp. grew superficially on sporulating galls and invaded the teliospores, while the *Tr. roseum* caused necrosis of significant segments of the galls. The high prevalence of these species at many weed sites called for a more intensive study of their impact on *U. morrisii*, particularly on its spread. It is unlikely that the mycoparasites were imported from Australia with the original *U. tepperianum* teliospores as a series of inoculations with spores, collected from healthy galls, were carried out in the glasshouse before collection for field releases. It was postulated that fungal species from similar galls caused by *Ravenelia* species on native *Vachellia* species were the potential source of the mycoparasite infections.

## Discussion

Invasive species threaten the existence of native and endangered species and reduce biodiversity. The economic cost of invasive species over the last 40 years (1970-2017) was estimated at US$1.3 trillion globally (Diagne et al., 2021). As of 2017, Australia spent or incurred losses from invasive species totaling approximately US$299 billion, with the costs of invasive plants totaling US$152 billion (Bradshaw et al., 2021). A substantial portion of the costs are allocated to management strategies, including prevention, eradication, and mechanical, chemical, and biological control to minimize the threats posed by invasive weeds (Culliney, 2005). Classical weed biocontrol becomes the only possible control strategy for invasive weeds when mechanical and chemical (herbicide) control strategies become economically too expensive, ineffective, or environmentally unacceptable (Charudattan, 2001; Culliney, 2005; Schwarzländer et al., 2018).

Thirty-one of the 100 worst invasive species are plants (Global Invasive Species Database; (http://www.iucngisd.org/gisd/100_worst.php). Over 80% of these invasive plants are under some form of classical insect and fungal biocontrol, including *Lantana camara* L., *Lythrum salicaria* L., *Miconia calvescens, Miconia crenata* (Vahl.) Michelang. (Syn: *Clidemia hirta* (L.) D. Don)*, Mikania micrantha, Reynoutria japonica* (syn.: *Fallopia japonica*), *Salvinia molesta* D. Mitch., and *Ulex europaeus* L. (Winston et al., 2021). However, the results of classical biocontrol programs are often variable. Less than 25% of released agents are having a ‘heavy impact’ on the target weed, resulting in significantly reduced needs for other control measures or making them no longer necessary (Hershenhorn et al., 2016; Schwarzländer et al., 2018). While insects remain the preferred classical biocontrol agents against invasive weeds, the use of plant pathogens, in both classical and inundative approaches, has significantly increased over the last 40 years since the first formal attempt for classical control with pathogens in the late 1960s (Charudattan, 2001; Hershenhorn et al., 2016; Morin, 2020; Winston et al., 2021).

Of the 36 fungal agents intentionally introduced, successful agents include the rust fungus *Puccinia chondrillina* Bubák & Syd. released on *Chondrilla juncea* L., the smut fungus *Entyloma ageratinae* released against *Ageratina riparia* (Barton et al., 2007), bridal creeper rust fungus *Puccinia myrsiphylli* released against *Asparagus asparagoides* (Morin et al., 2002) and the rust *Puccinia spegazzinii* on *Mikania micrantha* (Pollard et al., 2021). According to Schwarzländer et al. (2018), over 20% of fungal pathogens released as biocontrol agents had a consistently ‘heavy impact’ on the target weed, while the impact of a further 60% was medium or variable (low, medium, or heavy, depending on release site). However, quantitative data documenting their impacts is often limited, with the majority of reports of significant impact either anecdotal or subjective (Winston et al., 2021).

We reviewed the evidence that inadvertent biocontrol of introduced plant pathogens by naturally occurring fungal antagonists (endophytes and mycoparasites) contributes to the failure or variability of fungal weed biocontrol. We identified five main challenges that are presented by antagonists:

1) Antagonists reduce the sporulation of the fungal agent and damage spores, leading to reduced inoculum during production and reduced impact in the field (Morris, 1999; Morin et al., 2000a; Currie et al., 2020);
2) Antagonists impede maintaining healthy cultures of the agent, which interferes with safety and efficacy experiments in the lab or glass house (Morin et al., 2000a; Seixas et al., 2005; Seixas et al., 2007; Anderson et al., 2010; Tanner et al., 2015; Anderson et al., 2016);
3) To avoid culture contamination with antagonists, more resources need to be invested during agent collection and production, contaminated material needs to be discarded, and methods need to be developed to clean and maintain clean cultures and inoculum (Morin et al., 2000b; Barton et al., 2005; Anderson et al., 2010; Kurose et al., 2012; Anderson et al., 2016);
4) Antagonists reduce infection pressure of the pathogen in the field. By inhibiting the invasion of plant tissue, the production and viability of spores and the damaging of rust galls, the biocontrol effectiveness of the agent is reduced (Morris, 1999; Dodd et al., 2010; Kurose et al., 2012; Garbelotto et al., 2019);
5) Presence of antagonists affect laboratory, glasshouse, and field studies. This makes assessments of the biocontrol effectiveness and safety difficult (Ellison et al., 2004; Ellison et al., 2008).

The success or failure of an introduced insect biological control agent often depends on its rate of mortality from disease, predation, and hyperparasitism (Paynter et al., 2017). Similar to that scenario, predicting whether a fungal agent is likely to experience enemy-release or endophyte-release due to the absence of antagonists could assist agent prioritization, potentially making biocontrol both environmentally safer and more effective. Consequently, it may prove useful to determine the level of impact of mycoparasites and endophytes in the native range of fungal weed biocontrol agents, as a potential predictor of their impact on their target weeds (Paynter et al., 2010).

## Conclusion

From the mostly anecdotal evidence for inadvertent biocontrol of introduced plant pathogens, we conclude that naturally occurring fungal antagonists (endophytes and mycoparasites) may well contribute to the ‘perceived’ lack of success of fungal biocontrol agents. Antagonistic interference with fungal biocontrol agents should therefore be considered when planning future fungal weed biocontrol programs. The lack of evidence highlights the need for the collection and publication of quantifiable data, such as plant-associated and hyperparasitic microbial taxa. Investigations of the antagonist’s mode of action, host range and response to abiotic factors will improve our understanding of interactions between antagonists, fungal agents, and target plants. Antagonist-specific assessments (in the native and introduced range) will, as a consequence, improve the effectiveness of future biocontrol programs. Successful biocontrol of weeds using introduced fungi requires the introduced plant pathogen to suppress the invasive populations of the target weed. If mycoparasites or endophytes commonly interfere with the effectiveness of beneficial, intentionally introduced, plant pathogens, this will also be of interest to researchers seeking to intentionally control harmful plant pathogens.

## Author contributions

All authors participated in manuscript drafting, and the final editing was done by ADB and CL. All authors contributed equally to the article and approved the submitted version.

## Funding

Funding was provided through the Strategic Science Investment Fund, New Zealand Ministry of Business, Innovation and Employment.

## Conflict of interest

The authors declare that the research was conducted in the absence of any commercial or financial relationships that could be construed as a potential conflict of interest..

## Publisher’s note

All claims expressed in this article are solely those of the authors and do not necessarily represent those of their affiliated organizations, or those of the publisher, the editors and the reviewers. Any product that may be evaluated in this article, or claim that may be made by its manufacturer, is not guaranteed or endorsed by the publisher.
