# Supplementary material for: Plant pathogens as introduced weed biological control agents: Could antagonistic fungi be important factors determining agent success or failure?
Source: Front Fungal Biol. 2022 Jul 26;3:959753. doi: 10.3389/ffunb.2022.959753 (PMC10512343; doi:10.3389/ffunb.2022.959753)
Supplement: Supplementary file 1 [file Table_1.docx]

Supplementary Material

# Supplementary Data

**Mechanisms of Action**

**Fungal Endophytes**

Endophytes can be transmitted vertically (directly from parent to offspring) through the host tissue, seed, or vegetative propagules from one generation to the next or horizontally (among individuals) external to host tissues, by spores (Tadych et al., 2014). Except for grasses, most endophytes appear to be transmitted horizontally.

Generally there are four functional groups of endophytic fungi (Rodriguez et al., 2009) and these include :

**Class 1 Clavicipitaceous endophytes** are primarily vertically transmitted, with maternal plants passing fungi on to offspring via seed infections (Saikkonen et al., 2002). Colonized plants usually have a single dominant fungal isolate/genotype. They increase plant biomass, confer drought tolerance, produce toxic chemicals to animals and decrease herbivory (Clay, 1988). The benefits conferred are often dependent on the host species, host genotype, and environmental conditions (Saikkonen et al., 1998; Faeth and Sullivan, 2003; Faeth et al., 2006). They are found in grasses only and enhance the resistance of hosts to insect herbivory, confer tolerance to drought, and increase plant biomass (Rodriguez et al., 2009).

The highly diverse group of **Nonclavicipitaceous (NC)-endophytes** represent a polyphyletic assemblage of primarily ascomycetous fungi (any fungus in which the spores form inside an ascus) with diverse and often poorly defined or unknown ecological roles. The three functional classes are based on host colonization patterns, transmission mechanisms, *in planta* biodiversity levels, and ecological function (Rodriguez et al., 2009).

**Class 2 NC-endophytes** grow in both above- and below-ground tissues and are capable of extensive tissue colonisation, having limited diversity within individual host plants. They are vertically and horizontally transmitted and are able to confer habitat-adapted fitness benefits to host plants. They occur in high infection frequencies in plants growing in high-stress habitats. They increase host shoot and root biomass through the induction of plant hormones by the host plant or biosynthesis of plant hormones by the fungi. These endophytes protect their hosts to some extent against fungal pathogens through the production of secondary metabolites, fungal parasitism, or induced systemic resistance. The plants host defenses are not activated unless the host is exposed to plant pathogens (Rodriguez et al., 2009).

**Class 3 NC-endophytes** occur primarily or exclusively in above-ground tissues forming highly localized infections. They are highly diverse within host populations, plants, and tissue, and are horizontally transmitted. They potentially confer benefits or costs on host plants that are not necessarily habitat-specific, and are implicated in enhanced disease resistance, herbivore deterrence, changes in sensitivity to drought and other abiotic stressors. These endophytes inhabit diverse ecological roles including protection from plant pathogens, mutualisms, and antagonists (Rodriguez et al., 2009).

**Class 4 NC-endophytes (dark septate endophytes [DSE])** are primarily ascomycetous fungi that are either conidial or sterile. They are restricted to roots where they form melanized structures such as inter- and intracellular hyphae and microsclerotia. They are often associated with mycorrhizal fungi and capable of extensive tissue colonization, while their level of diversity within individual plants is insufficiently evaluated. They are neither host nor habitat-specific and ubiquitous in occurrence and abundant across various ecosystems. They are most prevalent in high-stress environments (Rodriguez et al., 2009).

**Mycoparasites**

Mycoparasitic interactions are either necrotrophic or biotrophic (Karlsson et al., 2017).

**Necrotrophic mycoparasites** are fungi that invade and kill their hosts. They have a broad host range and a less-specific mode of action. Primarily applied in field and greenhouse trials as potential biocontrol agents for crop diseases, the genera *Trichoderma* and *Clonostachys* are among the most studied mycoparasites. Their classification is based on the level of interaction between the parasite and its host (Moore et al., 2017), which may be through **contact necrotrophs** having hypha-to-hypha interference or **invasive necrotrophs** where the parasitic hyphae penetrate the host's hyphae.

**Biotrophic mycoparasites** establish a balanced relationship, with the mycoparasite growing on the still-living mycelium of the host fungus. The relationships are defined as i) **intracellular biotrophs** where the complete parasite thallus enters the host through the host cytoplasm and absorbs nutrients directly from it; ii) **haustorial biotrophs** where the haustoria formed by the parasitic hyphae penetrate the host hypha, and iii) **fusion biotrophs** where specialised contact cells accomplish direct cytoplasmic continuity with the host through fine pores in the hyphal walls forming inter-hyphal channels.

**References**

Clay, K. (1988). Fungal endophytes of grasses: A defensive mutualism between plants and fungi. *Ecology* 69(1)**,** 10-16. doi: <https://doi.org/10.2307/1943155>.

Faeth, S.H., Gardner, D.R., Hayes, C.J., Jani, A., Wittlinger, S.K., and Jones, T.A. (2006). Temporal and spatial variation in alkaloid levels in *Achnatherum robustum*, a native grass infected with the endophyte *Neotyphodium*. *Journal of Chemical Ecology* 32**,** 307–324. doi: <https://doi-org.landcareresearch.idm.oclc.org/10.1007/s10886-005-9003-x>.

Faeth, S.H., and Sullivan, T.J. (2003). Mutualistic asexual endophytes in a native grass are usually parasitic. *The American Naturalist* 161(2)**,** 310-325. doi: 10.1086/345937.

Karlsson, M., Atanasova, L., Jensen, D.F., Zeilinger, S., Heitman, J., James, T.Y., et al. (2017). Necrotrophic mycoparasites and their genomes. *Microbiology Spectrum* 5(2). doi: 10.1128/microbiolspec.FUNK-0016-2016.

Moore, D., Robson, G.D., and Trinci, A.P.J. (2017). *Chapter 16: Fungi as pathogens of animals, including man* [Online]. Available: <http://www.davidmoore.org.uk/21st_century_guidebook_to_fungi_platinum/Ch15_02.htm> [Accessed 29 April 2022].

Rodriguez, R.J., White Jr., J.F., Arnold, A.E., and Redman, R.S. (2009). Fungal endophytes: diversity and functional roles. *New Phytologist* 182(2)**,** 314-330. doi: 10.1111/j.1469-8137.2009.02773.x.

Saikkonen, K., Faeth, S.H., Helander, M., and Sullivan, T.J. (1998). Fungal endophytes: A continuum of interactions with host plants. *Annual Review of Ecology and Systematics* 29**,** 219-243. doi: 10.1146/annurev.ecolsys.29.1.319.

Saikkonen, K., Ion, D., and Gyllenberg, M. (2002). The persistence of vertically transmitted fungi in grass metapopulations. *Proceedings of the Royal Society B: Biological Sciences* 269(1498)**,** 1397-1403. doi: 10.1098/rspb.2002.2006.

Tadych, M., Bergen, M.S., and White Jr., J.F. (2014). *Epichloë* spp. associated with grasses: new insights on life cycles, dissemination and evolution. *Mycologia* 106(2)**,** 181-201. doi: 10.3852/106.2.181.
